# Effect of metformin on the eradication of H.Pylori infection in 25 -75 years old patients referring Loghman Hakim Hospital

**DOI:** 10.22088/cjim.13.3.567

**Published:** 2022

**Authors:** Shahriar Nikpour, Mohammad Salehi, Sina Homaee, Farnaz Saberian, Saeid Kalbasi

**Affiliations:** 1 Internal Ward, Loghman Hakim Hospital, Shahid Beheshti University of Medical Sciences, Tehran, Iran; 2 Internal Ward, Loghman Hakim Hospital, Shahid Beheshti University of Medical Sciences, Tehran, Iran; 3 Department of Internal Medicine, Shahid Beheshti University of Medical Sciences, Tehran, Iran

**Keywords:** Helicobacter pylori eradication, Metformin, Helicobacter pylori infection

## Abstract

**Background:**

Various ways of treating H.pylori infection are reported, such as triple-therapy and quadruple therapy for two weeks. Some side effects have been seen during these treatments, besides Helicobacter pylori becoming resistant to these antibiotics easily. According to some studies, there is a relationship between metformin and reduction in Helicobacter pylori infection. Thus, in this study, we determine the effects of metformin on Helicobacter pylori infection.

**Methods:**

We performed this assessment in a randomized, case-controlled way in the diagnosis of Helicobacter pylori infected outpatients and inpatients. In both groups (case group and control group), patients took two tablets for a two-week period. In the case group, the patients were given two metformin tablets (each containing 500mg of metformin (extended release) and in the control group, they were given two placebo tablets (each containing 500mg of white flour). We took h.pylori Ag stool test and rapid urease test to confirm the presence of Helicobacter pylori infection.

**Results:**

In this study, at first all the patients had positive h.pylori Ag stool test or positive rapid urease test. At the end of this study, the results of h.pylori Ag stool*- test presented that Helicobacter pylori infection was negative in 82.7% of the case group patients and 76% of control group patients which illustrates suppression of Helicobacter pylori infection. However, comparing to the control group (P=0.36), this difference was not statistically remarkable.

**Conclusion:**

According to these findings, it is stated that having metformin along with prescribed antibiotics can help decrease Helicobacter pylori infection.

A*mong all of the world’s population, about half of the human congregation is infected with Helicobacter pylori* considered as one of the most prevalent pathogens contaminating human beings. Except for cases with convincing limitations, we should cure all H. pylori infected patients. In the 90s decade, scientists started publishing different national and international instructions for the management of H. pylori-related infections and illnesses and periodically updated these guidelines regarding hints for treatment, diagnostic methods, and preferred abstinences during treatment (1). The infection caused by h.pylori is known as the primary reason of gastritis, peptic ulcer disease and gastric cancers. Approximately 89% of all gastric cancers are often attributed to H. Pylori infection. Previous studies have demonstrated that the infection of gastric epithelial cells caused by h.pylori, induced an epithelial–to–mesenchymal transition, which leads to the advention of cells with characteristics of a cancer stem cell (2).

Gastric adenocarcinoma is the fifth most common cancer after lung, breast, colorectal and prostate cancers and has a poor prognosis with only one in five patients surviving longer than 5 years after diagnosis (3). At first, this bacterium was categorized as a carcinogen of group I. Its pathogenesis is due to several virulence-associated factors (3). Diseases are alternatingly associated with the virulence markers of this bacterium. The best option to effectively cure h. pylori- associated illnesses is to suppress h. pylori in infected patients (4). Because of the increase in antibiotic resistance, standard treatment methods for curing Helicobacter pylori infection are becoming less effective these days. Specifically, the rapidly rising resistance of this pathogen to clarithromycin, levofloxacin, and metronidazole has led to recommendations against the empirical use of these drugs as part of combination therapies. Now, investigators report efficacy and safety findings for a metformin-based regimen (5). The rising number of subjects being target for H. pylori and the physiological and pharmacoeconomic charges of a second course of treatment have made gastroenterologists and microbiologists continue the search for new therapies (8). The therapies other than antibiotics used to suppress H. pylori infection is summarized in this review. Probiotics, phytomedicines and antioxidants, as therapies other than antibiotics, have been increasingly studied as potential replacements for the treatment of H. pylori (6). Furthermore, this therapy is accompanied by a higher rate of side effects. Thus, H.pylori eradication still remains imperfect and new treatment opportunities should be continually sought (6).

Metformin is a biguanide family molecule which is able to regulate the glucose metabolism. Indeed, metformin is an old molecule, prescribed in Type 2 diabetes and used extensively in clinical practice since the 1950s (7) Since2005, this molecule has been intensively studied for its antitumoral properties in different types of cancer (8) and for its ability to target the cancer stem cells, including gastric cancer (9) Moreover, metformin has been recently described to modify the gut microbiota of diabetic patients treated with this molecule (10). In 2018, a study stated that metformin had direct antimicrobial effects on H.Pylori in rats in vivo and invitro, but so far no study has been done on its antimicrobial effects in human samples (11).

Due to the low complications, safety and the availability and cheapness of metformin, we decided to investigate its antimicrobial effects on eradication of h.pylori. Our work is indeed the first to demonstrate a direct antimicrobial effect of metformin on a bacterium, opening potentially new roads to treat. H. pylori infected patients, and showing that this molecule has not yet revealed its full potential. We demonstrated that metformin could have a direct bactericidal effect on H. pylori.

## Methods

This study was double blinded randomized clinical trial study, during March 2018 and May 2020, on a patient who referred to Loghman Hospital as outpatients and inpatients due to gastrointestinal symptoms such as bloating, abdominal pain, nausea, and vomiting, or patients who underwent endoscopy and biopsy due to scope indication. They underwent stool Ag test diagnostic methods or rapid urease test, respectively, and thus h.pylori infection was confirmed in them.

Then these patients were divided into two groups, group A (case group) ؛received standard triple therapy amoxicillin 1gr every 12 hours, 1gr metronidazole every 12 hours, and 20 mg omeprazole twice daily before breakfast, and 30 minutes before dinner plus metformin extended release 500 mg twice daily (after lunch and dinner) for 14 days. Group B) controlled gr؛ (oupreceived standard triple therapy plus placebo capsules containing 500 mg white flour that is similar in appearance to metformin tablets twice daily for 14 days . To confirm that the intervention result on eradication of h.pylori infection with co-variate was evaluated. All of the patients that were eligible to enter the study had inclusion criteria and participated in the study voluntarily; and received a letter of satisfaction from the patients. After completing the questionnaire (including personal information and demographic information) they got eradication treatments of h.pylori infection and were evaluated. All demographic information and specifications included age, sex, underlying disease, type of treatment and monitoring were recorded for h.pylori infection eradication. Negative result of h.pylori Ag on stool test has defined as eradication criteria of h.pylori infection four weeks after treatment. considering that the similar study had not been done on human society so far, we were not able to determine the percentage difference between metformin users and people who did not use metformin, we compared the first 10 patients as a pilot study in two groups and based on a difference in the results obtained with α=0.05 and β=0.2 the sample size of 150 patients was obtained. 150 patients were consisted in this study (80 females, 70 males, mean age 41 years, range 25-75 years). Cases had symptoms such as uninvestigated dyspepsia, predominantly chronic or recurrent pain in upper abdomen, which suggests that organic disease requiring an endoscopic evaluation. This study was conducted over one-year period (between March 2018 and May 2020) in double-blinded RCT study, the patient had referred to Loghman Hospital after diagnosing h.pylori infection who had inclusion criteria and letter of satisfaction entered the study. An identical gastroenterologist performed all of the endoscopies employing a fiber endoscope. The admitted patients based on the reception order were randomly partitioned into 2 groups: i) the first group named group A (n=75) with a prescription of the standard triple therapy together with metformin; and group B (n=75) with a prescription of the standard triple therapy together with placebo.

Inclusion criteria: inpatients and outpatients 25-75 years old referred to Loghman Hospital with h.Pylori infection that confirmed with stool Ag test and /or rapid urease test. Exclusion criteria: diabetic patients, patients with contraindication for metformin such as GFR<45ml/min and liver failure and CHF Class III, IV and previous users of the metformin and h.pylori treatment eradication, those who suffered from PUD and gastrointestinal malignancy in past medical history in themselves and their families were from the study.

**Diagnosis of H. pylori infection:** We performed an endoscopy of the upper gastrointestinal tract of some patients. A nodular figure in the gastric mucosa was remarked as a support for h. pylori infection. 2 biopsy specimens were obtained from the antrum, and 2 from the corpus. We took biopsy specimens from areas with abnormal mucosa in the endoscopically suspected gastric inflammation patients. We performed a rapid urease test (Helident, RTA, Kocaeli, Turkey) on one of the biopsy specimens, and fixed the other specimens in 10% formalin solution. Then stained the slides with hematoxylin-eosin and Giemsa, and performed a histopathological examination to determine the h. pylori infection. Detection of a nodular appearance in the upper gastrointestinal tract endoscopy, positive rapid urease test, and the detection of h. pylori in the histopathological examination was established as the diagnosis of h. pylori infection. The study included patients facing at least 2 of these criteria.

**Diagnosis of H. pylori antigens in stool samples:** To assess the existence of h. pylori antigens in the stool samples the rapid, single step h. pylori card test (a qualitative immunochromatographic assay for the designation of h. pylori in stool samples) was performed. We evaluated the stool samples by the card test according to the manufacturer's protocol. The emergence of a single red band across the central window in the site marked with the control line was considered negative and the emergence of a red band both in the site marked with the result line and in the site marked with the control line was considered positive. The non-attendance of the control band in total, regardless of the appearance of the result site was considered invalid.

**Patient compliance and side effects:** Side effects such as any intolerance including abdominal pain, nausea, vomiting, constipation, belching, taste problems, lack of appetite, and diarrhea that would mandate the discontinuation of therapy were recorded at days 0, 7, 14. The records were analyzed by the same author who was uninformed about the treatment assignment.

**Treatment:** Group A received standard triple therapy, amoxicillin 1gr every 12 hours, 1gr metronidazole every 12 hours, and 20 mg omeprazole(twice daily before breakfast, and 30 minutes before dinner for 14 days) plus metformin 500 mg extended release twice daily for 14 days. while groupB (controlled group) received standard triple therapy plus capsules containing 500mg white flour that are similar in appearance to metformin tablets twice daily. Pockets containing pills were coded A and B by a non-researcher before the investigation, because the lack of informing the researcher from the received tablets by any of both groups is observed. The treatment duration was 14 days. For 28 days, after the fulfillment of the therapy, we performed h.pylori Ag on stool and UBT test to evaluate the success of therapy. Before participating in the study, an informed consent was obtained from the patient, and the study was carried out with the approval of the Ethics Committee of Shahid Beheshti University, School of Medicine. 

**Statistical analysis:** We used the IBM SPSS statistics for Windows Version 20.0 (IBM Corp, Armonk, NY, USA) in the statistical analysis, and analyzed the data using chi-square test and independent-sample t-test. P<0.05 were considered remarkable.

## Results

**Figure 1:**
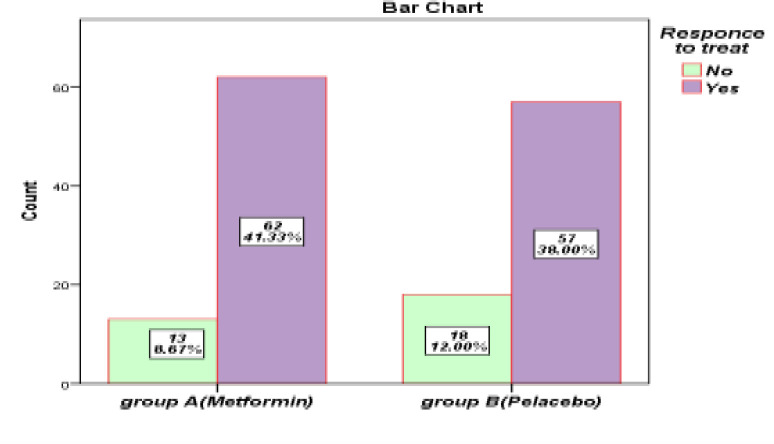
Comparison of two treatment groups A (metformin diet regimen) and B (placebo regimen) for response to treatment (chi-square analysis)

**Figure 2:**
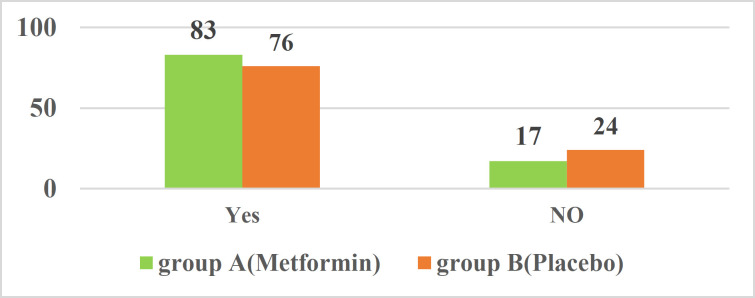
Comparison of response to treatment in two groups of four-drug treatment with metformin (group A) and four-drug treatment with placebo (group B)

The patients had h.pylori infection confirmed by stool Ag test diagnostic methods and/or rapid urease test and were divided in two groups .The patients in the case group (group A) took metformin tablet (two tablets each containing 500 mg of metformin extended release) for two weeks whereas the patients in the control group (group B) took two placebo tablets (each containing 500 mg of white flour) at the same time. This treatment regimen was taken for 2 weeks. 4 weeks after the completion of treatment for the evaluation of h.pylori eradication, the patients checked out with hpylori Ag on stool test in the same laboratory at Loghman Hospital. Study deviation of the age criterion of the participants in investigation was obtained 10.10±57.41.80 (53.3%) included females. Only 29(19.9%) patients were smoking. All the descriptive information of the patients appears in table 1. Treatment response status was assessed in both groups (table 2, figure 1). In group A (four-drug treatment with metformin) the rate of positive response to treatment was 82.7 % (62) and in group B (four-drug treatment with placebo) the rate of positive response to treatment was 76% (57) (Figure 2). In this study, chi-square test was used to investigate the relationship between positive response to treatment and qualitative variables under study, and independent t-test was used to examine the relationship between response to treatment and age as the only quantitative variable in the study (table 3). In the chi-square analysis, 52.1% of patients who responded to treatment received four metformin treatments. Also, 58.1% of patients who did not respond to treatment received four placebo treatments. However, there was no statistically significant difference between the positive response to treatment and the type of treatment received (P=0.3). In this analysis between the positive response to treatment and patients' gender (P=0.1), smoking (P=0.9), abdominal pain (P=0.3), nausea (P=0.3), bloating (P=0.7) reflux (P=0.4) and diagnosis, there was no statistically significant difference in independent t-test analysis, likewise no statistically remarkable difference between the positive response to treatment and the age of patients (P=0.1). We used multivariate logistic regression analysis to predict the factors that can be effective in recovery and positive response to treatment (Table 4). 

In this analysis, by calculating the odds ratio (OR) and Confidence interval 95%, only two variables, no reflux (P= 0.04, OR: 1.9, 95% CI: 1.25-2.75)) and having abdominal pain (P= 0.02), OR: 0.78, 95% CI: 0.1-1.15) before treatment, predicted improvement and positive response to treatment. This means that patients who did not have reflux before treatment responded 1.9 times more often, and patients who did not have abdominal pain before treatment responded 22% less (i.e. having abdominal pain before treatment was a predictor). Has been a positive response to treatment).

**Table 1:** Patient descriptive information (H pylori infected)

	**Variable**		**Variable**
53 (35.3) 97 (46.7)	Vomiting N (%) Yes No	41.57 ±10.10	Age (Mean±SD)
68 (45.3) 82 (54.7)	Bloating N (%) Yes No	70 (46.7) 80 (53.3)	Sex N (%) Male female
57 (38) 93 (62)	Reflux N (%) Yes No	29 (19.3) 121 (80.7)	Smoking N (%) Yes No
54 (36) 96 (64)	Diagnostic method N (%) Fecal Pathology	103 (68.7) 47 (31.3)	Abdominal pain N (%) Yes No

**Table 2:** Treatment response status in groups A and B based on fecal antigen test after two weeks

**Frequency** **N (%)**	**Variable**
620 (82.7) 13(17.3)	Group A(triple treatment with metformin) Positive response to treatment Negative response to treatment
57(76) 18 (24)	Group A(triple treatment with placebo) Positive response to treatment Negative response to treatment

**Table 3:** Investigating the relationship between positive response to treatment and the variables under study

**P-value**	**Positive answer to treatment**		**Variable**
	**YES** **119 (79.3 %)**	**NO** **31 (20.7 %)**	
0.1	41.02± 9.76	43.67± 11.23	Age
0.1	52 (43.7) 67 (56.3)	18 (58.1) 13 (41.9)	sex Male female
0.9	23 (19.3) 96 (80.7)	6 (19.4) 25 (80.6)	Smoking Yes No
0.3	84 (70.6) 35 (29.4)	19 (61.3) 12 (38.7)	Abdominal pain Yes No
0.3	40 (33.6) 79 (66.4)	13 (41.9) 18 (58.1)	Nausea Yes No
0.7	53 (44.5) 66 (55.5)	15 (48.4) 16 (51.6)	Bloating Yes No
0.4	47 (39.5) 72 (60.5)	10 (32.3) 21 (67.7)	Reflux Yes No
0.08	47 (39.5) 72 (60.5)	24 (77.4) 7 (22.6)	Diagnostic method Fecal Pathology
0.3	62 (52.1) 57 (47.9)	13 (41.9) 18 (58.1)	Type of treatment Triple treatment with metformin Triple treatment with placebo

**Table 4:** Multivariate logistic regression analysis to predict treatment response in patients with Helicobacter pylori infection (statistically significant shown with *)

**P-.value**	**OR(95% CI)**	**Variable**
0.6	1.32 (0.45 – 3.82)	Age 40≤ (refrence group)
0.1	0.5 (0.19 – 0.65)	Sex male Female (refrence group)
0.9	1.01 (0.33 – 3.10)	Smoking NO YES (Refrence group)
0.02*	0.78 (0.1 – 1.15)	Abdominal pain NO YES (Refrence group)
0.2	2.80 (0.53 -16)	Nausea NO YES(Refrence group)
0.2	3.33 (0.43 – 20)	Bloating NO YES (Refrence group)
0.04*	1.9 (1.25 – 2.75)	Reflux NO YES (Refrence group)
0.09	3.09 (0.82 – 11.57)	Diagnostic method pathology Stool (refrence group)
0.3	1.66 (0.62 – 3.44)	Type of treatment Group A (Metformin) Group B (Placebo)

## Discussion

At the end of the study, the result of Helicobacter pylori stool antigen assay among the case group and after taking metformin was more favorable than the control group, although this difference was not significant. The results of fecal antigen test demonstrated that H. pylori infection in metformin users eradicated the bacterium in 82.7% of cases, but this result was not statistically significant compared to the control group. Recent studies have shown that Helicobacter pylori reduces PTEN expression by inducing promoter methylation, and metformin reduces PTEN promoter gene methylation induced by CagA and PTEN MRNA expression, and HCG-27 cell proliferation and apoptosis.

 A study by Courtois Sarah et al. in November 2018 at the University of Boreaux in France found that metformin could inhibit the growth of Helicobacter pylori on mouse models in vivo and in vitro. In a study by Behrens et al.with a regimen of omeprazole, amoxicillin and clarithromycin for 2 weeks, eradication of H. pylori was observed in 83% of cases (12). Walch et al. have also launched a regimen of clarithromycin and metronidazole for one week eradication of 90% as reported (13). In Iran, Malekzadeh et al. reported a two-week diet consisting of amoxicillin, bismuth, Ranitidine, and furarazolidone eradicating Helicobacter pylori in 82% of cases reported in this group, However, the present study reported the eradication of this bacterium in the metformin group of 82.7%.) According to studies, an acceptable diet in the treatment of this bacterium should be associated with 85_90% success (14) because several studies have shown that the use of a treatment regimen with a higher rate of eradication reduces reinfection by Helicobacter pylori, although many cases of re-infection of this bacterium are recurrences of previous infections (15,16). A study by Tireno et al. reported the potency of omeprazole, amoxicillin, and clarithromycin in 75% eradication of Helicobacter pylori over two weeks (17, 18). A study by Hojo et al., the eradication rate of Helicobacter pylori with amoxicillin, metronidazole, bismuth and omeprazole was reported at 76% (19). In the present study, the eradication rate of Helicobacter pylori in the control group with the treatment regimen including the three types of antibiotics (amoxicillin, omeprazole, metronidazole and placebo) was 76%. 

In the present study, there was no significant relationship between metformin use and eradication of Helicobacter pylori. A 2018 study by Chin-Hsiao Tseng et al. at Taiwan University Hospital Center found that patients treated with metformin decreased the incidence of Helicobacter pylori depending on the dose. In the present study, treatment failure was reported in 17% in the case group and 24% in the control group. Studies have shown that during the first period of treatment for Helicobacter pylori, treatment failure is seen in an average of 5-12% of cases (19). One of the important reasons for this is the rapid resistance of this bacterium to antibiotics. High resistance has been reported for metronidazole, up to 95% (20). In most studies in Iran, Helicobacter pylori resistance to this antibiotic has been reported in the range of 60 to 70% (21). The present study showed that not only Helicobacter pylori not resistant to metformin but also the use of metformin with antibiotics induces a double effect on the eradication of this bacterium. In the present study, patients who did not complain of abdominal pain had a 22% lower response to treatment than the group who complained of abdominal pain. Also, the lack of reflux was another predictor item that patients without reflux had higher response to treatment (1.9 times) than the group who complained of reflux. 

In the outcome of this study, the suppression of Helicobacter pylori infection was represented by the negative results of h.pylori Ag stool*- assay in 82.7% of the case group and 76% of control group. Nevertheless, in comparison with the control group, the difference was not remarkable (P= 0.36). As a result, the investigations show that taking metformin along with prescribed antibiotics can help in reducing Helicobacter pylori infection.
